# Reliability of Different RBC Indices and Formulas in Discriminating between β-Thalassemia Minor and other Microcytic Hypochromic Cases

**DOI:** 10.4084/MJHID.2015.022

**Published:** 2015-02-20

**Authors:** Elahe Bordbar, Mehdi Taghipour, Beth E. Zucconi

**Affiliations:** 1 MD, General practitioner, Shiraz University of Medical Sciences; 2 PhD, Department of Pharmacology & Molecular Sciences, Johns Hopkins School of Medicine

## Abstract

**Objective:**

Different indices and formulas of CBC parameters have been suggested as indicators of early stage screenings to detect couples with β-thalassemia minor (BTMi). In this study, we evaluated the accuracy of five previous published formulas and compared them to our new formula (│80-MCV│×│27-MCH│) in screening of β-thalassemia.

**Methods:**

All couples in the premarital β-thalassemia screening program of Roodbar, Iran, for whom molecular analysis had been done, were selected during two years. The red blood cell parameters were applied to each formula, and a ROC curve was plotted for each one to check its discriminative effectiveness in β-thalassemia detection.

**Result:**

None of the studied indices and formulas demonstrated 100% precision. However, we found that the Shine–Lal formula and our formula had the highest sensitivity in identifying BTMi individuals. The highest specificity belonged to our formula and Sirdah formula.

**Conclusion:**

Previous studies reported different sensitivities and specificities for the formulas. This can be attributed to different kinds of HBB gene mutations in various populations. Undoubtedly, physicians in different areas should evaluate the accuracy of published formulas for their own populations in the discrimination of BTMi from other causes of microcytic hypochromic anemia.

## Introduction

β-thalassemia major is a disaster that affects both patients and their β-thalassemia carrier parents. β-thalassemia is widespread from the Mediterranean area to Southeast Asia. Approximately 1.5 % of world’s population are carriers of the associated genetic mutation and 60000 symptomatic individual born annually^1^ In Iran, the gene frequency of β-thalassemia is high and greatly variable in different areas.^2^ The highest rate of the carriers reported was about 10% around both the Caspian Sea and the Persian Gulf. In other areas the prevalence is between 4% and 8%.^3^ About 3–5 % of Iranian population is heterozygous for the *HBB* gene.^4^

Carriers of β-thalassemia are usually clinically asymptomatic.^5^ However, they have typical characteristics of microcytic and hypochromic anemia in their laboratory data. Their CBCs show mean corpuscular volume (MCV) less than 80 fl and mean corpuscular hemoglobin (MCH) less than 27 pg.^6^ Although carriers seem healthy, if both parents are β-thalassemia carriers, one-fourth of their children may develop β-thalassemia major according to pure Mendelian genetics.^7^ Prenatal diagnosis is a feasible policy to prevent the birth of β-thalassemia major newborns.^8^–^10^ This strategy requires a powerful screening test for β-thalassemia traits to identify couples at risk of having a fetus with β-thalassemia major. Because screening should be done for all who want to conceive, the method should be simple and highly sensitive.^11^ Mass screening programs, especially in developing countries where resources are limited, should be cost effective.

Due to low cost of checking CBC, red cell parameters have been used as first indicators of possible β-thalassemia minor (BTMi).^4^ Not only have MCV and MCH values been shown to be highly inaccurate predictors of parental genotype, but also multiple cut off points (between 70–80 for MCV and 20–30 for MCH) have been reported.^12^ This mandates the development of more accurate indices and formulas. Since the early 1970s, different indices and formulas of CBC parameters have been suggested as easy and inexpensive tools to determine whether an individual is in danger of developing BTMi or not. (England & Fraser, 1973; Mentzer, 1973; Lafferty et al., 1996). However these formulas have various accuracies reported in different studies.^13^

In this study we want to evaluate the accuracy of some previous published formulas and our new formula (│80-MCV│×│27-MCH│) to differentiate BTMi from other causes of microcytic hypochromic anemia in Iranian population.

## Materials and Methods

The samples of this study were collected from couples who were referred for the premarital screening program of β-thalassemia in Roodbar, Iran, a city located south of Kerman. This screening program was started about 20 years ago under full control and supervision of the Iranian Ministry of Health.^14^ The method of screening was described in details in previous studies.^15^

For the present study all couples, to whom molecular analysis had been done and the exact genotype was confirmed (no mutation, β, α or sickle cell mutations), were selected from Roodbar since April 2011 till March 2013. According to their medical records, the CBC parameters were recorded from the first CBC documented in their medical charts.

We used the following cell counter–based formulas in our study:

F1, Shine and Lal Index: MCV × MCV × MCH/100;
F2, Mentzler Index: MCV/RBC count;
F3, Srivastava Index: MCH/RBC count;
F4, Ehsani formula: (MCV - 10×RBC)
F5, Sirdah formula: (MCV- RBC - 3 ×Hb)
F6, Our formula: │80-MCV│×│27-MCH│

The red blood cell (RBC) count, MCH, hemoglobin (Hb) and MCV were applied to each formula. Normality of data was checked using SPSS software version 15.0. If the data had a normal distribution, parametric analyses (student t-test) were done. Sensitivity and specificity, positive predictive value (PPV), negative predictive value (NPV) and accuracy were calculated for each index and formula. Receiver operating characteristic (ROC) curves were plotted for each index and formula to check discriminative efficacy, derive new cutoffs, and select the best indices and formulas for β-thalassemia detection. Statistically significant differences were defined as comparisons resulting in p<0.05.

## Results

Data of 504 partners (252 men and 252 women) were collected. Among them 353 (70%) individuals were put into non-BTMi group (278 had defects in α genes and 75 had no gene defects in the molecular study) and 151(30%) were identified as being defective in the *HBB* gene. None were previously diagnosed as thalassemia carriers. In our study, 44/504 (8.7%, 28 males, 16 females) cases had MCV >80 fl (range 80–91) and MCH<27 pg. Of these 44, only three of them were diagnosed as BTMi carriers. On the other hand, none of the 23/504 (4.5%, 14 males, 9 females) individuals with MCH>27 pg (range 27–29.8) and MCV<80 fl had BTMi.

**Figure 1:**
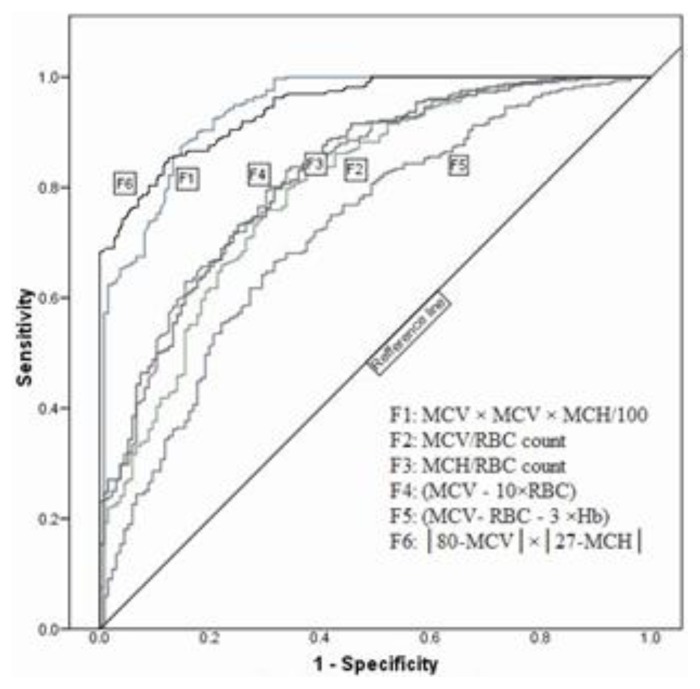
ROC curve of formulas.

Means of all RBC parameters, except RBC counts, were higher in non-BTMi group compared to the BTMi group. Table 1 summarizes the mean ± standard deviation of the various hematological parameters obtained from individuals with microcytic and/or hypochromic anemia. Also, the results of applying the six different prediction formulas are shown. The Student *t* test showed significant differences in all above RBC parameters and HbA2 levels (*P* < 0.05). However, no significant difference in the levels of HbA and HbF of individuals with or without β thalassemia was observed. Furthermore, all of these six formulas can differentiate BTMi from non-BTMi with different accuracies. Some of these data did not have normal distribution; therefore, non-parametric analyses were performed to confirm the parametric analysis results (p<0.001) (Table 1). ROC curves were plotted (Figure 1) to obtain new cut-off points with higher sensitivity and specificity for every formula and parameter, as depicted in Table 2. The optimal cut-off point was obtained according to area under the curve (AUC) and maximum of sensitivity + specificity. The indicted criterion value is the cut-off value corresponding to the highest accuracy (max [sensitivity + specificity]). As indicated in Table 2, none of the indices studied demonstrated 100% precision in the recognition of BTMi. However, the Shine–Lal and Sirdah formulas demonstrated the highest (87.6%) and lowest (66%) sensitivity, respectively. The highest and lowest specificities were derived from our formula (87.9%) and Ehsani formula (70%), respectively. We found the largest AUC in our formula (0.943) which is obviously greater than other formulas. The second largest is the AUC of Shine-Lal formula which is 0.940. The smallest AUC belongs to Sirdah formula (0.720).

Based on the result, the best cutoff point in our formula is >44.76. Therefore, when the MCV and MCH of an anemic patient are applied to our formula, it predicts the BTMi with higher accuracy compared to when the cutoff point is 44.76. According to the best cut off point for each formula in our study, the performance of each one was calculated. Although the best sensitivity belonged to the Shine–Lal formula (87.6%), the sensitivity of our formula was 84.7%. The best specificity (84.9%), PPV (75.1%), NPV (93%) and accuracy (86.9%) belonged to our formula (Table 3).

## Discussion

Six formulas were applied to each case. The two best formulas were that of Shine and Lal and our formula, which demonstrated 87.6% and 84.7% sensitivity, and 84.7% and 87.9% specificity, respectively. Our formula with discrimination of 44.76 showed to be a reliable formula to differentiate BTMi from non BTMi causes of microcytic and hypochromic anemia. While comparing these two formulas to others, the accuracy is highly plausible. It is completely congruent with previous studies in which the Shine and Lal formula was identified as the best method to detect BTMi.^2^,^16^–^18^ The cause of these differences is fundamental to the formula. The data show that BTMi individuals had lower MCV and MCH compared to patients with other causes of microcytic hypochromic anemia.^19^ Therefore, by multiplying MCV by MCH, the differences become much more obvious. Only these two formulas have this characteristic. As is seen in Table 1, although all RBC parameters can differentiate BTMi from non-BTMi, if a new formula needs to differentiate BTMi from other causes of microcytic hypochromic anemia, several parameters should be multiplied by each other to increase the strength of differentiation. Also these formulas, including multiple factors, may be less erroneous than MCV or MCH measurements alone.^2^ Our result showed that MCH is slightly more accurate than MCV in discriminating individuals with BTMi, as suggested by a previous study.^20^ However, another study claimed that MCV=80 is a proper parameter for screening.^21^

Previous studies reported different sensitivities and specificities for the formulas we mentioned in this study.^2^,^13^,^16^ The difference may be due to diverse methodology of the studies. Some previous studies^13^ categorized patients to two groups, one with definite diagnosis of BTMi and the other with iron deficiency anemia (IDA). This means that they excluded other causes of microcytic hypochromic anemia such as α-thalassemia trait, chronic diseases, and rare causes such as lead poisoning and sideroblastic anemia; but it is clear that the mean MCV and MCH of individuals with the α-thalassemia trait is higher than that of those with BTMi,^22^ so omitting this group can increase sensitivity and specificity of the formulas.

A practical formula with high sensitivity and specificity is essential for physicians to rely on their laboratory results to discriminate between BTMi and other causes of microcytic hypochromic anemia, specifically in premarital counseling and screening. Therefore, studies should include other causes instead of IDA to help physicians do further workups for their patients.

The Batebi et al.^2^ study was compatible with our study in considering other causes of microcytic hypochromic anemia rather than IDA. However, they reported higher sensitivity and specificity for Shine–Lal (83.1%–90.6%) and Mentzler formulas (86.3%–85.4%). Furthermore, the mean values of Shine–Lal (BTMi=1359.80, non-BTMi=1988.91) and Mentzler formulas (BTMi=10.98, non-BTMi=15.03) calculated by this study are different from our study (Table 1). Pornprasert et al. reported that the accuracy of formulas were varied in different populations.^23^ Hence, It is important for physicians of different populations to establish their own formulas or evaluate the published formulas to discriminate BTMi from other causes of microcytic hypochromic anemia.^13^

In this study the ROC curve is generated to evaluate different indices and formulas. The formula will be more accurate and reliable when the area under the ROC curve is greater. The AUC of 1.0 represents the best differentiation while AUC of 0.5 represents the least valuable one.^13^ Collectively, the AUC of all previous formulas except Shine-Lal formula were smaller than the AUC value reported by Sirdah et al. This is also true about sensitivity and specificity. The optimal cut-off point (criterion value) we obtained according to AUC is compatible with previous published cut-off values for all formulas except for the Shine-Lal formula. However, approximately all cut off values we observed are the same as that of Sirdah et al. study.

The distribution of *HBB* gene mutation is not random all over the world and in each population it has a specific distribution.^24^ The inter-population differences in effects of various RBC indices and mathematical formulas in discrimination of BTMi and non-BTMi can be attributed to variable mutation spectrums of the thalassemia disease in different populations.

The MCV level of individuals with BTMi are associated with the severity of anemia; it represents that different mutations in *HBB* gene correlate with different MCV levels.^13^,^25^ Moreover, previous studies showed that hematological phenotypes are associated with the type of mutation in individuals with BTMi which could explain the inter-population differences.^13^,^26^

All mean values of RBC parameters (MCV, MCH, MCHC, Hct, Hb) we analyzed were lower in the BTMi group compared to the non-BTMi group except for RBC count, in agreement with the results of Batebi et al. The result will be completely different when the comparison is done between BTMi and IDA as reported by Sirdah et al. We found that HbA2 (α_2_δ_2_) level can significantly differentiate BTMi from non-BTMi; however, no significant differences are found in the levels of HbA (α_2_β_2_) and HbF (α_2_γ_2_) in sera from these two groups. If the mutation occurs in one β gene (BTMi), the production of β chain will decrease in the serum but δ and γ chain production will increase to compensate β chain insufficiency. Therefore, HbA2 and HbF levels will increase but HbA level will decrease. If a mutation occurs in one or more α genes, production of α chain will decrease and all HbA, HbA2 and HbF serum levels will decrease but a percentage of them remain normal. Hence, we expect to find significant differences in these types of Hb between BTMi and non-BTMi individuals.

## Conclusion

Conclusively, the performance of this new index is slightly better than that was obtained by Shine and Lal formula. It was portrayed in our observation that none of the differentiation indices or formulas provided 100% sensitivity or specificity for discriminating purposes. The cut-off points may be varied in different populations due to variable gene mutations or prevalence of other causes of anemia such as iron deficiency, folate or B12 deficiency that can change RBC parameters.

## Figures and Tables

**Table 1:** Mean ± standard deviation and P-value of the various hematological parameters obtained from individuals with microcytic and/or hypochromic anemia in BTMi and non-BTMi groups.

	Mean ± SD In BTMi (n=151)	Mean ± SD In non-BTMi (n=351)	P-value
**Age (yr)**	26.5 ± 7.17	26.4 ± 6.14	0.8
**Hb (g/dl)**	11.7 ± 1.59	13.0 ± 1.65	<0.001
**Hct (%)**	37.4± 5.26	40.4 ± 4.94	<0.001
**MCV (fl)**	66.2 ± 5.15	75.5 ± 3.90	<0.001
**MCH (pg)**	20.7± 1.57	24.3 ± 1.85	<0.001
**RBC × 10^12^/l**	5.69± 0.80	5.32 ± 0.73	<0.001
**MCHC (g/dl)**	31.2± 1.13	32.1 ± 1.77	<0.001
**HbA2 (%)**	5.0±0.74	2.6 ± 0.64	<0.001
**HbF (%)**	1.7±2.40	1.3 ± 0.95	0.3
**HbA (%)**	92.5±10.71	93.1 ± 11.45	0.6
**MCV^2^×MCH/100**	922.0±212.36	1399.5 ± 229.31	<0.001
**MCV/RBC**	11.9±2.08	14.5 ± 2.59	<0.001
**MCH/RBC**	3.7±0.65	4.7 ± 0.87	<0.001
**MCV-10×RBC**	9.3±10.46	22.3 ± 9.29	<0.001
**MCV-RBC-3×Hb**	25.5±7.04	31.0 ± 6.53	<0.001
**│80-MCV│×│27-MCH│**	94.0± 50.88	17.8 ± 18.81	<0.001

**Table 2:** Evaluation of different mathematical formulas in the differentiation of BTMi from non-BTMi.

	AUC (95% CI)	Previous published cut off value	Criterion value	sensitivity	specificity
**Shine–Lal formula** **MCV^2^×MCH/100**	0.940 (0.917–0.963)	< 1530	< 1110	87.6	84.7
**Mentzler formula** **MCV/RBC**	0.795 (0.750–0.840)	< 13	< 12.85	75.7	70.5
**Srivastava formula** **MCH/RBC**	0.823 (0.782–0.864)	< 3.8	< 4.17	73.4	75
**Ehsani formula** **MCV-10×RBC**	0.822 (0.781–0.863)	< 15	< 14.75	79.5	70
**Sirdah formula** **MCV-RBC-3×Hb**	0.720 (0.699–0.771)	< 27.0	< 28.64	66	86
**Our formula** **80-MCV│×│27-MCH│**	0.943 (0.923–0.963)	-	> 44.76	84.7	87.9

**Table 3:** Performance of various formulas in differentiating BTMi from other causes of microcytic hypochromic anemia.

	Sensitivity	Specificity	PPV	NPV	Accuracy
**Shine–Lal formula** **MCV^2^×MCH/100**	87.6	84.7	74.7	87.6	86.7
**Mentzler formula** **MCV/RBC**	75.7	70.5	54	86.4	74.2
**Srivastava formula** **MCH/RBC**	73.4	75	53.4	87.8	73.8
**Ehsani formula** **MCV-10×RBC**	79.5	70	58	86.7	76.7
**Sirdah formula** **MCV-RBC-3×Hb**	66	86	45.3	83.8	64.3
**Our formula** **│80-MCV│×│27-MCH│**	84.7	87.9	75.1	93	86.9

## References

[1] Galanello R, Origa R (2010). Beta-thalassemia. Orphanet J Rare Dis.

[2] Batebi APA, Esmailian R (2012). Discrimination of beta-thalassemia minor and iron deficiency anemia by screening test for red blood cell indices. Turk J Med Sci.

[3] Rahim F Correlation of beta-thalassemia mutations with alpha-thalassemia: an experience of the southwestern region of Iran. Hematology.

[4] Hashemizadeh H, Noori R Premarital Screening of Beta Thalassemia Minor in north-east of Iran. Iran J Ped Hematol Oncol.

[5] Leung TN, Lau TK, Chung T (2005). Thalassaemia screening in pregnancy. Curr Opin Obstet Gynecol.

[6] Yamsri S Genotype and phenotype characterizations in a large cohort of beta-thalassemia heterozygote with different forms of alpha-thalassemia in northeast Thailand. Blood Cells Mol Dis.

[7] Li D (2006). Detection of alpha-thalassemia in beta-thalassemia carriers and prevention of Hb Bart’s hydrops fetalis through prenatal screening. Haematologica.

[8] Kosaryan M (2009). Knowledge, attitude, and practice of reproductive behavior in Iranian minor thalassemia couples. Saudi Med J.

[9] Tongsong T (2000). Prenatal control of severe thalassaemia: Chiang Mai strategy. Prenat Diagn.

[10] Jaovisidha A (2000). Prevention and control of thalassemia in Ramathibodi Hospital, Thailand. Southeast Asian J Trop Med Public Health.

[11] Sirichotiyakul S (2005). Sensitivity and specificity of mean corpuscular volume testing for screening for alpha-thalassemia-1 and beta-thalassemia traits. J Obstet Gynaecol Res.

[12] Sirichotiyakul S (2009). A comparison of the accuracy of the corpuscular fragility and mean corpuscular volume tests for the alpha-thalassemia 1 and beta-thalassemia traits. Int J Gynaecol Obstet.

[13] Sirdah M (2008). Evaluation of the diagnostic reliability of different RBC indices and formulas in the differentiation of the beta-thalassaemia minor from iron deficiency in Palestinian population. Int J Lab Hematol.

[14] Karimi MMV, Mehrabanejad S, Mohaghegh P, Afrasiabi A, Dehbozorgian J, Silavizadeh S, Bazrafshan A (2012). Prevalence of Delta Beta Thalassemia Minor in Southern Iran. Iranian Journal of Blood and Cancer.

[15] Karimi M (2007). Premarital screening for beta-thalassaemia in Southern Iran: options for improving the programme. J Med Screen.

[16] Rathod DA (2007). Usefulness of cell counter-based parameters and formulas in detection of beta-thalassemia trait in areas of high prevalence. Am J Clin Pathol.

[17] Lafferty JD (1996). The evaluation of various mathematical RBC indices and their efficacy in discriminating between thalassemic and non-thalassemic microcytosis. Am J Clin Pathol.

[18] Madan N (1999). Red cell indices and discriminant functions in the detection of beta-thalassaemia trait in a population with high prevalence of iron deficiency anaemia. Indian J Pathol Microbiol.

[19] Rahim F (2009). Microcytic hypochromic anemia patients with thalassemia: genotyping approach. Indian J Med Sci.

[20] Karimi M, Rasekhi AR (2002). Efficiency of premarital screening of beta-thalassemia trait using MCH rather than MCV in the population of Fars Province, Iran. Haematologia (Budap).

[21] Alireza Moafi SV, Nikyar Zahra, Zeinalian Mehrdad, Momenzadeh Maryam, Rahgozar Soheila (2010). Prevalence of Minor β-thalassemia Based on RBC Indices among Final Suspected Individuals in Premarital Screening Program Referred to Genetic Laboratories. International Journal of Hematology Oncology and Stem Cell Research (IJHOSCR).

[22] Sankar VH (2006). Genotyping of alpha-thalassemia in microcytic hypochromic anemia patients from North India. J Appl Genet.

[23] Pornprasert S Red cell indices and formulas used in differentiation of beta-thalassemia trait from iron deficiency in Thai school children. Hemoglobin.

[24] Haghi MPN, Hoseinpour FMA, Hoseinpour F (2010). AA, beta thalassemia in Iran. Journal of Shaheed Sadoughi University of Medical Sciences.

[25] Rund D (1992). Mean corpuscular volume of heterozygotes for beta-thalassemia correlates with the severity of mutations. Blood.

[26] Rosatelli C (1992). Heterozygous beta-thalassemia: relationship between the hematological phenotype and the type of beta-thalassemia mutation. Am J Hematol.

